# Rabies immunoglobulin should not be forgotten in category III exposure

**DOI:** 10.1590/0037-8682-0470-2021

**Published:** 2021-12-17

**Authors:** Hasan Tahsin Gozdas

**Affiliations:** 1 Bolu Abant İzzet Baysal University, Faculty of Medicine, Department of Infectious Diseases and Clinical Microbiology, Bolu, Turkey.

Dear Editor:

I read with considerable interest the article by Duarte *et al*.^1^ entitled “Clinical aspects of human rabies in the state of Ceará, Brazil: an overview of 63 cases,” recently published in *Revista Brasileira de Medicina Tropical*. However, I want to highlight an important point regarding the administration of rabies immunoglobulin.

The authors stated that most bites in the case of their patient cohort were on the hands (21; 42%) and head (9; 18.4%). It is well-known that suspected rabid bites on the hands and head are category III rabies exposure. Accordingly, in these patients, rabies vaccination and rabies immunoglobulin should be administered according to World Health Organization guidelines^2^. 
